# Two-year prognosis of multinodular goiter following radiofrequency ablation based on all nodule burdens

**DOI:** 10.1530/ETJ-23-0134

**Published:** 2024-02-19

**Authors:** Rui Guo, Bowen Zheng, Tao Wu, Yufan Lian, Tinghui Yin, Yuting He, Jingya Qin, Zhicheng Yao, Wen Xu, Jie Ren

**Affiliations:** 1 Department of Medical Ultrasonics, The Third Affiliated Hospital of Sun Yat-sen University, Guangdong Province Key Laboratory of Hepatology Research, Guangzhou, Guangdong, People’s Republic of China; 2 Department of General Surgery, The Third Affiliated Hospital of Sun Yat-sen University, Guangdong Province Key Laboratory of Hepatology Research, Guangzhou, Guangdong, People’s Republic of China; 3 Department of Endocrinology and Metabolism, The Third Affiliated Hospital of Sun Yat-sen University, Guangdong Province Key Laboratory of Hepatology Research, Guangzhou, Guangdong, People’s Republic of China

**Keywords:** radiofrequency ablation, nodular goiter, efficacy, retreatment

## Abstract

**Objective:**

Few studies use all nodule burdens to specify the prognosis of multinodular goiter (MNG) following radiofrequency ablation (RFA), so this study addresses this question for MNG after completely ablating dominant nodules.

**Methods:**

The RFA indications for MNG include 2–5 benign nodules with over 50% normal tissue on ultrasound, 1–3 well-defined benign dominant nodules on cytology, largest diameter ≥20 mm and/or with clinical complaints, and patient refusal or unable to undergo surgery. A retrospective study of 185 MNG patients with completely ablated dominant nodules in a single-session RFA was conducted. The efficacy and complications were evaluated at 1, 6, 12 months, and yearly thereafter. Based on retreatment risks, progressive disease (PD), stable disease (SD), and complete relief (CR) were introduced to assess all nodule load changes. PD was clarified as having new/non-target nodules that newly appeared to ACR TI-RADS≥4, or new/enlarged non-target nodules ≥1 cm.

**Results:**

The initial ablation ratios of target nodules were 100% at one month. During a mean 22.38 ± 13.75 months (range, 12–60 months), the volume reduction rate of ablated nodules was 98.25% at 24 months without regrowth. Cosmetic and symptomatic scores decreased to 1 and 0, respectively, after 48 months. Of the patients, 9.7% (18/185) had PD and the retreatment rate was 2.2% (4/185). The complication rate was 2.7% (5/185).

**Conclusion:**

RFA provides cosmetic and symptomatic relief for an average of two years. RFA is a useful minimally invasive treatment modality for selected MNG patients.

## Introduction

Thyroid nodules (TNs) are commonly detected via high-resolution ultrasonography (US) and affect approximately 70% of the general population (1, 2). Although most are benign TNs (BTNs) and asymptomatic, a noticeable but small percentage will progressively enlarge and cause cosmetic and/or symptomatic problems necessitating surgical resection (3, 4, 5). However, surgical interventions for TNs are somewhat disadvantageous in that the risks of trauma, scar formation, and mild to severe complications are high, as is the prevalence of hypothyroidism. As a result, nonsurgical and minimally invasive interventions like radiofrequency ablation (RFA) have been widely used and recognized as highly effective and safe treatment options for BTNs in many authoritative guidelines (6, 7, 8, 9, 10).

However, it is well recognized that over half of the patients with BTNs are MNG, which presents as two or more nonpalpable or palpable nodules within an enlarged thyroid (11). This condition poses some challenges for RFA (9, 10), because, unlike surgery, which allows the removal of the entire lobe or gland in MNG patients, RFA is only indicated for the treatment of well-defined dominant nodules of MNG according to current guidelines (9, 10). Although four clinical trials have shown favorable results on the shrinkage of ablated dominant nodules in MNG patients (12, 13, 14, 15), evidence supporting the efficacy of RFA in improving cosmetic outcomes and relieving symptoms associated with whole thyroid swelling or all nodule burdens in a large sample remains scarce. In addition, considering that the condition of non-target nodules may change and new nodules may appear, possible retreatment may be inevitable. However, to our knowledge, no systematic study has focused on the actual magnitude of this problem, and little is known. Given our experience, we hypothesized that RFA might be clinically efficacious for MNG patients whose dominant TNs were completely ablated. If this is proven to be the case, this may suggest the possibility of RFA for selected suitable MNG patients.

Therefore, in this retrospective study, we evaluated the prognosis of MNG based on all nodule burdens for patients whose dominant nodules were completely ablated in a single session of RFA.

## Materials and methods

### Institutional guidelines for RFA and patient selection

**Figure 1:**
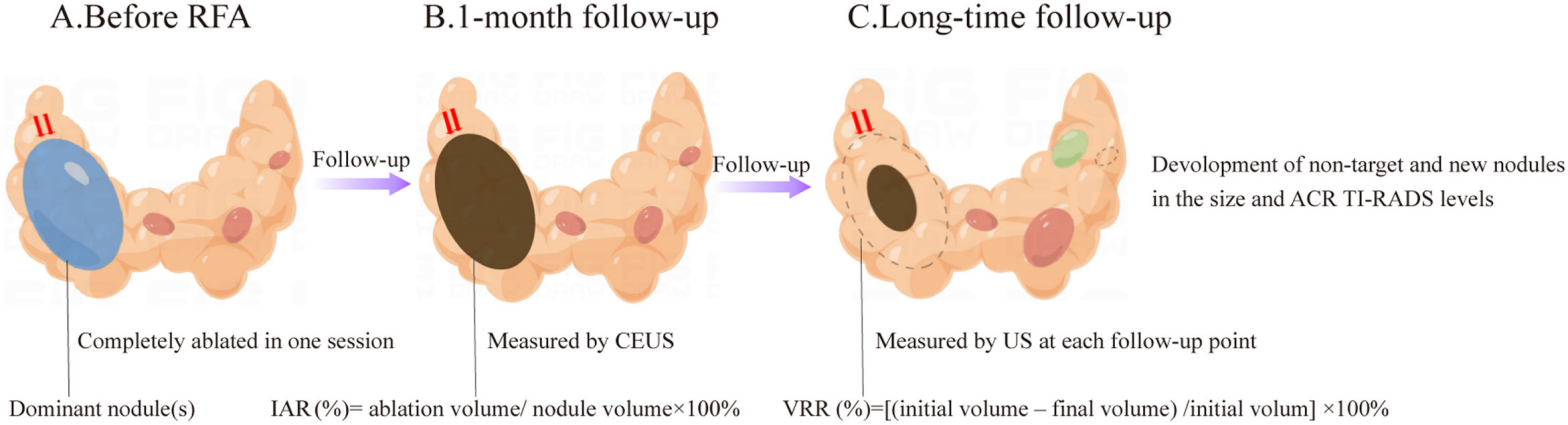
Diagram of the indications of RFA for MNG and observed indicators during the follow-up. (A) In the thyroid, the total number of nodules was less than five and at least 50% of normal tissue was left. All thyroid nodules had a benign appearance on ultrasound (ACR TI-RADS ≤3). RFA was performed for the dominant target nodules (red arrows), which were confirmed as Bethesda class II on cytology, with a largest diameter ≥20 mm and/or complaints of symptomatic or cosmetic problems, and the remaining small benign nodules (ACR TI-RADS ≤3 and ≤20 mm) except for dominant target nodules were non-target nodules (red nodules). (B–C) At 1 month, the IAR of ablated dominant nodules was measured by CEUS (red arrows). During the follow-up, the development of all thyroid nodules, including the VRR (red arrows) of ablated dominant nodules and the development of non-target (red nodules) and new nodules (green nodule), was monitored. The schematic was drawn by Figdraw. ACR TI-RADS, American College of Radiology Thyroid Imaging, Reporting and Data System; CEUS, contrast-enhanced ultrasonography; IAR, initial ablation ratio; RFA, radiofrequency ablation; US, ultrasound; VRR, volume reduction rate.

This retrospective study was approved by the Ethics Committee of the Third Affiliated Hospital of Sun Yat-sen University (no. (2015)2-215), and all patients provided written informed consent for treatment. In our institute, by combining many guidelines for thermal ablation (6, 7, 8, 9, 10), the indications for MNG patients to receive RFA were as follows: (i) with a total of two to five nodules and at least 50% normal tissue left (Fig. 1A); (ii) with benign-appearing of all TNs on ultrasound (ACR TI-RADS ≤3); (iii) with no intra-thoracic extension on CT or MR imaging; (iv) with one to three well-defined dominant nodules (Fig. 1A, blue nodule), which were confirmed as Bethesda class II on cytology, with a largest diameter ≥20 mm and/or complaining of cosmetic or symptomatic problems; and (v) refusal or unable to undergo thyroidectomy due to underlying diseases. The following were contraindications for RFA: (i) severe cardiopulmonary insufficiency and coagulation disorders; (ii) nodules with massive calcifications.

**Figure 2:**
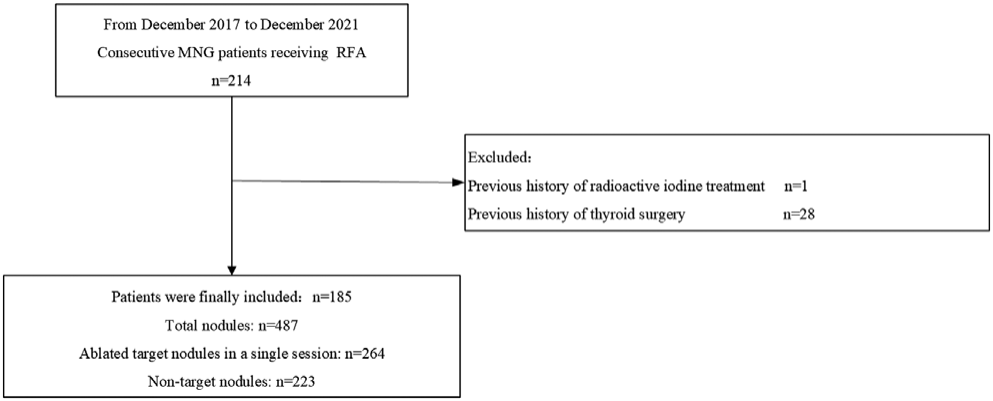
Flowchart of the retrospective study. MNG, multinodular goiter; RFA, radiofrequency ablation.

Between December 2017 and December 2021, all 214 consecutive patients with MNG who qualified for the above criteria underwent the single-session RFA at the Third Affiliated Hospital of Sun Yat-sen University, and their complete medical records were reviewed. We included subjects who fulfilled the following criteria: (i) complete ablation of all the dominant nodules in one session (The initial ablation ratios (IAR) were 100% confirmed by contrast-enhanced US (CEUS) at 1 month after RFA); (ii) no high-risk factors of thyroid cancer, including a family history of thyroid cancer, a history of hereditary syndromes indicating a predisposition to thyroid cancer, or a history of radiation exposure to the head and neck in childhood (16); (iii) no previous history of thyroid surgery or radioactive iodine treatment; and (iv) underwent more than 1 year of follow-up. Based on our selection criteria, 29 patients were excluded due to their previous history of thyroid surgery or radioactive iodine treatment. Ultimately, 185 patients were included (Fig. 2).

### Pre-RFA evaluation

All patients were subjected to US examination, clinical assessment, and thyroid function tests before RFA. Once fine-needle aspiration biopsy was performed to confirm the benignity of all target dominant nodules. The number, location, composition, size, and ACR TI-RADS score of all TNes were determined via US examination. The composition of the nodule was classified in terms of the proportion of its solid component (17): (i) >75%, solid; (ii) 25%–75%, mixed; and (iii) <25%, cystic. The total volume of the thyroid gland included the left lobe, the right lobe, and the volume of target dominant nodules in the isthmus (18). Moreover, in the clinical assessment, the patients were asked to provide their cosmetic grading scores and symptomatic grading scores. The cosmetic score is assessed by radiologists using the following scale (6): (i) no visible or palpable mass, (ii) no cosmetic problem but palpable mass, (iii) cosmetic problem on swallowing only, (iv) a readily detected cosmetic problem. The symptom score was obtained by a questionnaire concerning five clinical symptoms (13): compression, cough, difficulty swallowing, voice change, and pain. Each positive symptom was allocated 1 point, so that the symptom scores ranged from 0 to 5.

### RFA procedure

When we ablated multiple nodules, to avoid causing serious damage to the recurrent laryngeal nerves, all the dominant nodules in the ipsilateral thyroid lobe were ablated first, starting with the largest nodule, until all the suitable dominant nodules of the unilateral thyroid lobe were completely ablated sequentially. In subsequent observation, the radiologist providing treatment subjectively assessed the patients’ voices for 10 min to determine the functional integrity of the recurrent laryngeal nerve of the treated side. Ablation of the dominant nodules in the contralateral lobe was performed in the same manner.

One radiologist with 7 years of experience completed all RFA procedures using the VRS01 RFA system (STARmed, Korea) and initiated internal cooling of the 18G electrodes with 10 mm or 7 mm active tips under the guidance of the same US equipment. Local anesthesia, a lateral approach, a moving-shot technique, and a hydrodissection approach (5% glucose) were used during the RFA treatment.

In particular, CEUS was performed preoperatively, intraoperatively, and immediately postoperatively to identify the active tissue of the dominant nodules. If there were areas of enhancement in the ablated nodule during the RFA procedure, supplementary ablation guided by CEUS was performed until the whole target nodule was completely ablated.

### Follow-up evaluation

For follow-up, patients underwent periodic US examination, clinical assessment, and thyroid function tests at 1, 6, 12 months after RFA and then yearly thereafter. All US examinations were performed by three or four experienced radiologists, and another radiologist subsequently analyzed both cross-sectional and longitudinal dynamic images of the whole thyroid gland at each visit and included nodules with a largest diameter ≥3 mm for analysis.

### Evaluation of response to treatment on ablated dominant nodules

Several parameters were systematically collected to evaluate the response to RFA, as shown in Fig. 1. The IAR was associated with technique efficacy, defined as the ratio between the ablated volume and the volume of the ablated nodules (18). It was evaluated at 1 month by CEUS.

The volume reduction rate (VRR) was used to determine the extent of shrinkage of the ablated nodule (19) and was evaluated by US at each follow-up point. Regrowth of ablated dominant nodules was defined as the volume of the ablated nodules increasing by ≥50% compared to the minimum recorded volume measured during the follow-up (20, 21).

### Evaluation of resolution on clinical problems

In addition, the cosmetic and symptomatic scores of MNG patients were systematically recorded in the same manner during each visit from baseline until the last visit.

### Evaluation of development on all nodules

After complete ablation of the dominant nodules, when considering the need for retreatment due to the development of non-target nodules (remaining small benign nodules, ACR TI-RADS ≤ 3 and ≤ 20 mm) and/or new nodules, a comprehensive assessment of all nodules should be performed. Focusing on the risks of retreatment, we classified and defined all nodule burdens after RFA in three directions, as shown in Table 1.

**Table 1:** The definitions of the criteria about all nodule burdens for MNG after RFA.

	Definitions
PD^b^	Complete necrosis of ablated nodules
	and either
	non-target nodules enlarged^a^ to ≥1 cm with ACR TI-RADS ≤3;
	or
	new nodules developed to ≥1 cm with ACR TI-RADS ≤3;
	or
	non-target nodules or new nodules developed to ACR TI-RADS ≥4
SD	Complete necrosis of ablated nodules
	and either
	unenlarged non-target nodules with ACR TI-RADS ≤3
	or
	non-target nodules or new nodules developed to <1 cm with ACR TI-RADS ≤3
CR	Complete necrosis of ablated nodules without other nodules

^a^Enlargement of the non-target nodule was defined as a 20% increase in ≥2 diameters with a minimum increase of 2 mm or a 50% increase in volume; ^b^Qualifying criteria of PD may increase the risk of retreatment.

ACR TI-RADS, American College of Radiology Thyroid Imaging, Reporting and Data System; CR, complete relief; MNG, multinodular goiter; PD, progressive disease; RFA, radiofrequency ablation; SD, stable disease.

Progressive disease (PD) may increase the retreatment risk. It includes non-target nodules enlarged to ≥ 1 cm with ACR TI-RADS ≤3, or new nodules developed to ≥1 cm with ACR TI-RADS ≤3, which represent the trends of causing cosmetic and/or symptomatic problems; or non-target or new nodules developed to ACR TI-RADS ≥4.

Stable disease (SD) contained non-target or new nodules developed to <1 cm with ACR TI-RADS≤3, and these nodules developed relatively slowly and were small. Complete relief (CR) contained complete necrosis of all ablated nodules without any active nodules in the entire thyroid. Furthermore, when the MNG patients with non-target and/or new nodules showed PD and were deemed suitable for RFA, retreatment was considered.

### Evaluation of complications

Complications were recorded based on the criteria of the Society of Interventional Radiology (SIR) (22, 23). SIR classifications A–B were regarded as minor complications, and SIR classifications C–F were regarded as major complications.

### Statistical analysis

SPSS statistical software, version 25.0 (IBM Corp.), was used to perform the statistical analysis. Qualitative variables are expressed as numbers (percentages), and quantitative variables are expressed as medians (ranges) or means ± standard deviations. Quantitative variables were compared using t-test or Mann–Whitney *U* test, and qualitative variables were compared using Fisher’s exact test or the chi-square test. Two-sided* P*-values < 0.05 were considered statistically significant.

## Results

A total of 185 MNG patients (mean age, 43.48 years; 148 women and 37 men) with 487 nodules were included in the study, and the median follow-up period was 22 months (range, 12–60 months) as of the data cutoff. The demographic characteristics of all patients and nodules were summarized in Table 2. Before treatment, the initial cosmetic and symptomatic scores of these 185 patients were 4 (range: 1–4) and 1 (range: 0–4), respectively. To solve these problems, 264 dominant nodules (54.4%) with a maximum diameter of 30 mm (range, 20–66 mm) were completely ablated by single-session RFA. Of these, 114 (61.6%) patients had one ablated dominant nodule, 63 (34.1%) patients had two ablated nodules, and the remaining 8 (4.3%) patients had three ablated dominant nodules. The median ablation time and energy were 587 s (range: 51–3113 s) and 4.34 kcal (range: 0.47–19.35 kcal), respectively, and the ablation energy per nodule was 2.64 kcal (range: 0.21–16.89 kcal). After the procedure, 223 nodules (45.6%) with a median largest diameter of 5.9 mm (range: 3.0–18.8 mm) were non-target nodules, and 152 (82.2%) patients had ≥1 non-target nodule.

**Table 2:** Baseline characteristics of all 185 MNG patients and 487 nodules. Data are presented as mean ± S.D. or as median (range) or *n* (%).

Baseline characteristics	Values
Patients’ characteristics	
Age (years)	43.38 ± 13.78
Sex (Female)	148 (80.0%)
BMI (kg/m^2^)	22.10 ± 2.78
FT3 (pmol/L)	4.40 (3.01–8.21)
FT4 (pmol/L)	13.11 (9.34–22.62)
TSH (uIU/mL)	1.12 (0.01–6.39)
Follow-up time (months)a	22.38 ± 13.75 (12–60)
Underlying diseases	
Hypertension	9 (4.9%)
Diabetes	6 (3.2%)
Coronary heart disease	3 (1.6%)
Liver transplant	3 (1.6%)
Renal transplant	6 (3.2%)
Cosmetic score	4 (1–4)
Symptomatic score	1 (0–4)
Nodule characteristics	
Number of nodules per patient	
2	100 (54.1%)
3	58 (31.3%)
4	22 (11.9%)
5	5 (2.7%)
Ablated target nodule	
Number of nodules per patient	
1	114 (61.6%)
2	63 (34.1%)
3	8 (4.3%)
Maximum diameter (mm)	30 (20–66)
Volume (mL)	6.30 (0.64–59.50)
Left	114 (43.2%)
Right	130 (49.2%)
Isthmus	20 (7.6%)
Cystic	47 (17.8%)
Mixed	69 (26.1%)
Solid	148 (56.1%)
Ablation time (sec)	587 (51–3113)
Total energy delivered (Kcal)	4.34 (0.47–19.35)
Energy delivered per nodules (Kcal)	2.64 (0.21–16.89)
Non-target nodule	
Number of nodules per patient	
0	33 (17.8%)
1	93 (50.3%)
2	49 (26.5%)
3	8 (4.3%)
4	2 (1.1%)
Maximum diameter (mm)	5.8 (3.0–18.8)
Volume (mL)	0.05 (0.01–2.17)
Left	107 (48.0%)
Right	99 (44.4%)
Isthmus	17 (7.6%)
Cystic	46 (20.6%)
Mixed	126 (56.5%)
Solid	51 (22.9%)

MNG, multinodular goiter; BMI, body mass index; FT3, free triiodothyronine; FT4, free thyroxine; TSH: thyroid stimulating hormone. ^a^value in parentheses is the range.

### Treatment efficacy in VRR

**Figure 3:**
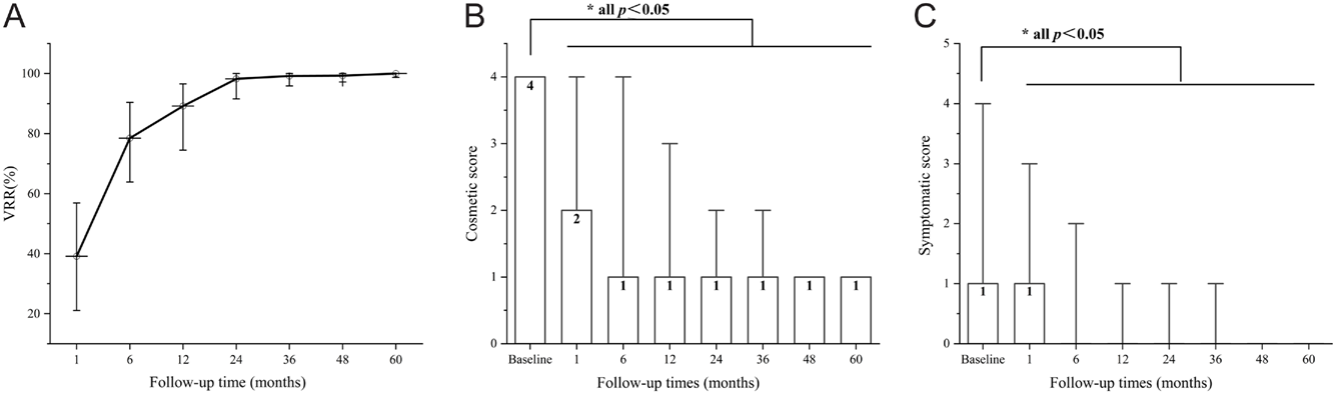
Trends of (A) VRR on ablated nodules, (B) cosmetic score, and (C) symptomatic score on MNG patients in the whole observation period. Compared with that at baseline, the cosmetic score and symptom score of MNG patients significantly decreased at 1-, 6-, 12-, 24-, 36-, 48- and 60 months, *P < 0.05. VRR, volume reduction rate.

The IAR of all target nodules was 100% confirmed by CEUS at 1 month. When we analyzed the trend of the ablated nodule volume after the procedure, significant shrinkage was observed during the follow-up period (*P* < 0.05). Specifically, the ablated nodule volume at one month was significantly lower than that at baseline (*P* < 0.05) and the median VRR achieved 39.25% (range: 0%–89.90%). Further significantly progressive VRR was seen during the first two years (*P* < 0.05), which was 98.25% (range: 37.27%–100%) at 24 months. Until 36 months, the nodule volume recorded did not significantly differ from that at 24 months and remained stable thereafter. The ablated nodule volumes at each ultrasound evaluation were presented in Table 3. The respective changes in VRR are shown in Fig. 3A. In particular, none of the ablated nodules regrew during the observation period.

**Table 3:** Treatment efficacy at each follow-up point. Data are presented as median (range).

	*n*	Volume of ADN (mL)	VRR (%)	Regrowth (%)	Volume of TG (mL)	Cosmetic score	Symptomatic score
Baseline	185	6.30 (0.64–59.50)	–	0 (0)	26.37 (7.87–127.91)	4 (1–4)	1 (0–4)
1 month	185	3.19 (0.25–32.66)^a^	39.25 (0–89.90)	0 (0)	18.38 (7.87–96.63)^a^	2 (1–4)^a^	1 (0–3)^a^
6 months	170	1.04 (0.03–22.31)^a,b^	78.47 (17.97–99.42)	0 (0)	15.10 (7.32–51.09)^a,b^	1 (1–4)a	0 (0–2)^a^
12 months	178	0.50 (0–18.21)^a,b,c^	90.90 (25.29–100)	0 (0)	12.48 (6.48–37.77)^a,b,c^	1 (1–3)a	0 (0–1)^a^
24 months	89	0.09 (0–8.44)^a,b,c,d^	98.25 (37.27–100)	0 (0)	11.17 (6.54–20.86)^a,b,c,d^	1 (1–2)^a^	0 (0–1)^a^
36 months	36	0.06 (0–4.19)^a,b,c,d^	99.20 (51.89–100)	0 (0)	10.20 (6.54–13.40)^a,b,c,d^	1 (1–2)^a^	0 (0–1)^a^
48 months	14	0.06 (0–3.77)^a,b,c,d^	99.31 (64.00–100)	0 (0)	9.56 (7.44–13.53)^a,b,c,d^	1 (1)^a^	0 (0)^a^
60 months	10	0.05 (0–0.67)^a,b,c,d^	99.33 (96.40–100)	0 (0)	8.87 (7.71–12.19)^a,b,c,d^	1 (1)^a^	0 (0)^a^

^a^*P* < 0.05 versus baseline; ^b^*P* < 0.05 vs 1 month; ^c^*P* < 0.05 vs 6 months; ^d^*P* < 0.05 vs 12 months.

ADN, ablated dominant nodules; IAR, initial ablation ratio; TG, thyroid gland; VRR, volume reduction rate.

### Treatment efficacy in cosmetic and symptomatic scores.

Both cosmetic and symptomatic scores. improved after RFA (Table 3 and Fig. 3). The cosmetic scores improved significantly from 4 at baseline to 2 at the 1-month assessment (*P* < 0.05), and all patients had a cosmetic score of 1 at 48 months after RFA (Fig. 3B). Similarly (Fig. 3C), the symptomatic scores were significantly improved at one month (*P* < 0.05) and no patient complained of any symptoms after 48 months.

### The development of all nodules

When we evaluated the development of all nodules based on the risk of retreatment (Table 4), we found that the vast majority of patients (90.3%, 167/185) had slow or no progression without a high possibility of retreatment. In particular, 22.2% of patients (41/185) showed the best development result, CR, in which no other nodules were presented within the entire thyroid. However, 9.7% of patients (18/185) developed PD and showed 25 enlarged, non-target nodules and new nodules measuring ≥1 cm with ACR TI-RADS ≤ 3, increasing the risk of the potential need for retreatment due to cosmetic and/or symptomatic problems.

**Table 4:** The development of unablated and new nodules after RFA.

	Patients (*n*, %)/ Nodules, *n*	Nodule type	Baseline	End point		
				ACR TI-RADS	Maximum diameter	Change in nodule size
PD	18 (9.7%)					
	16	Non-target	ACR TI-RADS ≤ 3	≤3	≥1 cm	With enlargement^a^
	9	New	Undetected	≤3	≥1 cm	–
SD	126 (68.1%)					
	151	Non-target	ACR TI-RADS ≤ 3	≤3	< 1 cm	Any
	30	Non-target	ACR TI-RADS ≤ 3	≤3	≥1 cm	Without enlargement^a^
	32	New	Undetected	≤3	<1 cm	–
CR	41 (22.2%)					
	26	Non-target	ACR TI-RADS ≤ 3	Undetected		

^a^The enlargement of the non-target nodule was defined as a 20% increase in ≥2 diameters with a minimum increase of 2 mm, or a 50% increase in volume.

ACR TI-RADS, American College of Radiology Thyroid Imaging, Reporting and Data System; CR, complete relief; PD, progressive disease; SD, stable disease.

**Figure 4:**
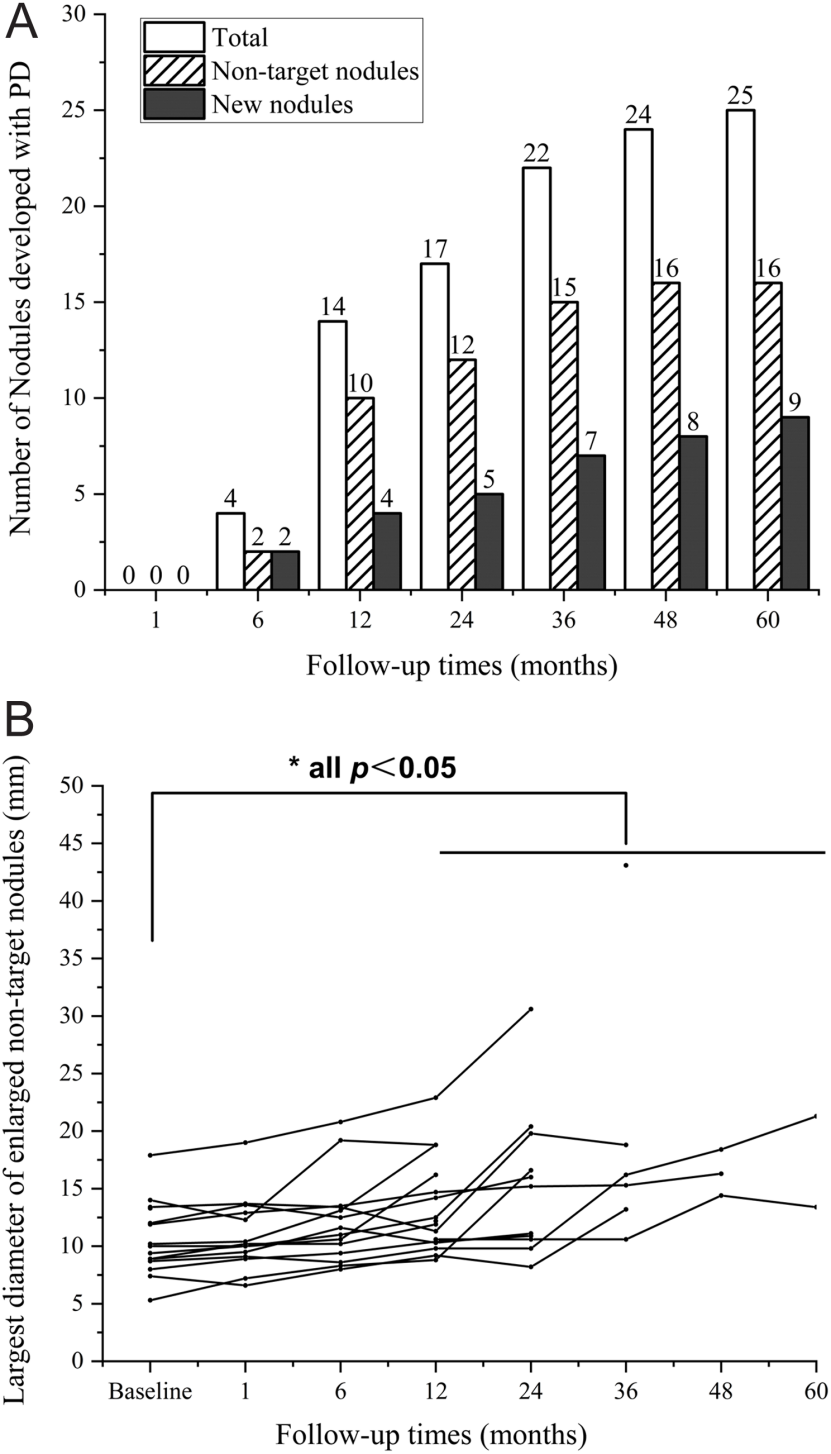
(A) The number of nodules developed with PD and (B) the largest diameter of 16 enlarged non-target nodules at each follow-up point. These non-target nodules were significantly enlarged in largest diameter at the 12-, 24-, 36-, 48-, and 60-month follow-ups compared with baseline, *P < 0.05. PD, progressive disease.

The details related to PD are presented in Fig. 4. With respect to the time of progression, it showed a trend of increase from fast to slow and continued over time. Specifically, most of the patients (61.1%, 11/18) with half of the nodules (56.0%, 14/25) progressed rapidly within the first year of follow-up. Gradual progression occurred during the subsequent four years. Considering the enlarged size of 16 non-target nodules, significant spontaneous enlargement from 9.7 mm (range: 5.3–17.9mm) at baseline to 17.4 mm (range: 13.4–21.3mm) was observed with the rates of 2.8 mm/year at 60 months. All nine new nodules developed to ≥1 cm with ACR TI-RADS ≤ 3. During the follow-up period, no non-target or new nodules developed to ACR TI-RADS ≥ 4.

### Retreatment

**Figure 5:**
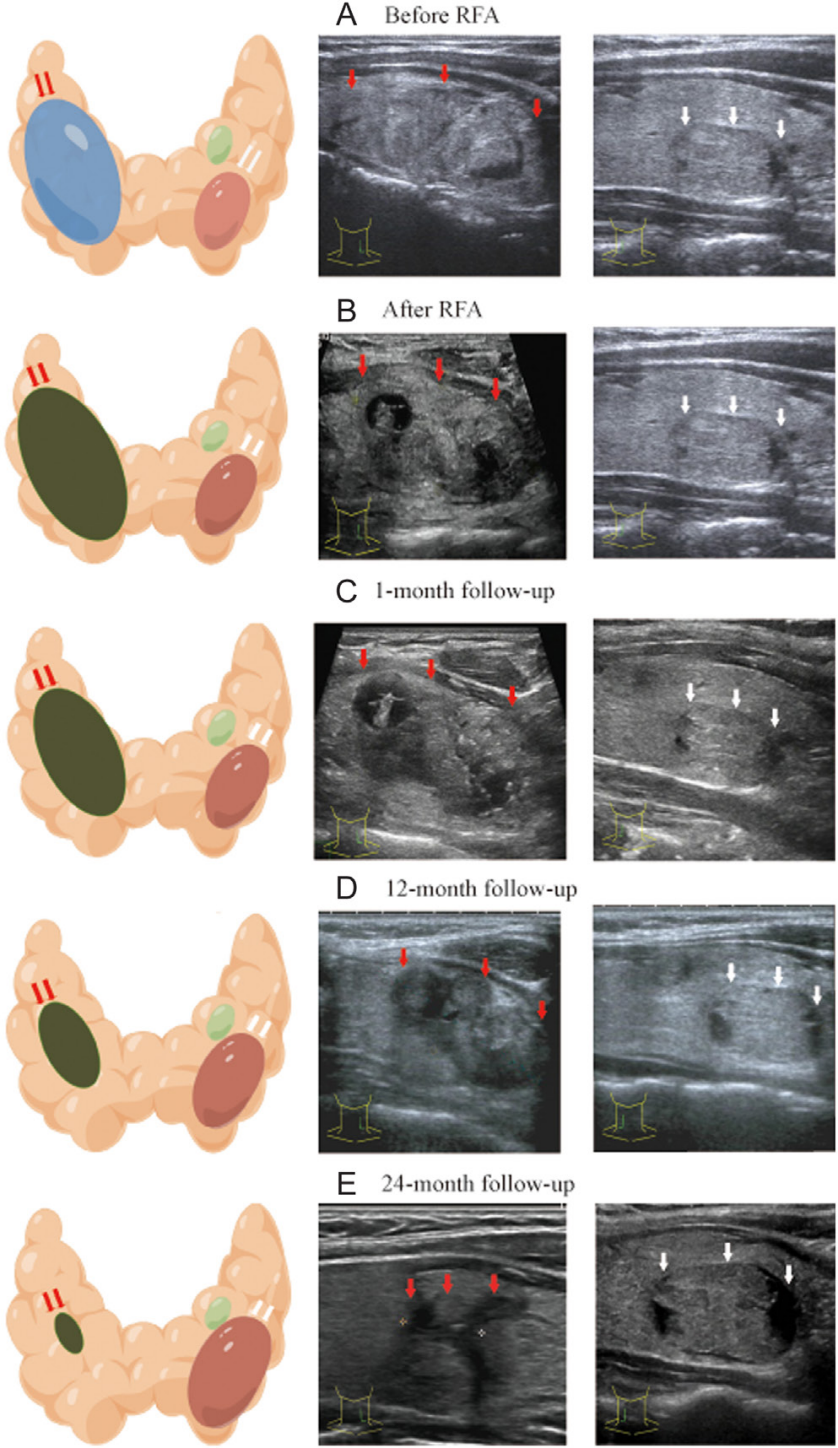
A case qualified for retreatment due to the PD of a non-target nodule. Female, 27 years, BMI 26.35 kg/m^2^, TSH 1.31 uIU/mL. (A) Ultrasonography before RFA showed three nodules in both lobes of the thyroid: one in the left lobe, size, 43 × 19 × 48 mm (red arrow); and the other two in the right, size, 17.9 × 9.9 × 11.7 mm (white arrow), and 8.0 × 6.0 × 5.8 mm. The patient received RFA to treat the dominant nodule in the left lobe, and the other 2 nodules were non-target nodules. (B) The ablated dominant nodule was completely ablated in one session (red arrow) which was confirmed by CEUS. (C–E) The ablated dominant nodule in the left lobe showed significant shrinkage during the follow-up (red arrow) and achieved a volume reduction rate of 97.80% at 24 months (red arrow). Both non-target nodules in the right lobe showed PD. The smaller lesion was enlarged from 8.0 × 6.0 × 5.8 mm at baseline to 10.3 × 5.9 × 10.9 mm at 24 months, and the larger one enlarged from 17.9 × 9.9 × 11.7 mm at baseline to 30.6 × 25.5 × 21.3 mm (white arrow) at 24 months causing cosmetic problems. This patient qualified the indications of retreatment due to this larger non-target nodule. The schematic was drawn by Figdraw.

A total of four patients (2.2%, 4/185) qualified for retreatment (Table 5) due to the PD of non-target nodules. A typical case was shown in Fig. 5.

**Table 5:** Characteristics of four patients qualified the indications of retreatment.

	Patient A	Patient B	Patient C	Patient D
Patients’ characteristics				
Age (years)	27	29	38	14
Sex	Female	Female	Female	Female
BMI (kg/m^2^)	26.35	28.28	21.48	16.94
Thyroid function status	Normal	Normal	Normal	Normal
Pregnancy during follow-up	Yes	No	No	No
Underlying diseases	No	No	Liver transplant	No
First treatment of DN				
*n*	1	2	2	2
Maximum diameter at baseline (mm)	48	52; 22	43; 25	39; 36
Volume at baseline (mL)	20.52	11.76; 1.87	26.46; 1.57	5.71; 7.16
Follow-up time (month)	24	60	24	36
VVR at endpoint (%)	97.80	96.23; 100	92.97; 100	95.90; 99.27
PD nodules^a^				
Nodules type	ENTN	ENTN	ENTN	ENTN
ACR TI-RADS at endpoint	3	3	2	2
Maximum diameter (mm)				
At baseline	17.9	8.7	9.4	13.3
At endpoint	30.6	21.3	20.4	43.1
Volume (mL)				
At baseline	1.09	0.18	0.21	1.00
At endpoint	8.70	2.39	1.86	13.17

BMI, body mass index; VRR, volume reduction rate; PD, progressive disease; ACR TI-RADS, American College of Radiology Thyroid Imaging, Reporting and Data System; DN, dominant nodules; ENTN, enlarged non-target nodule.

^a^these nodules developed PD and qualified for re-treatment.

### Complications

Five cases (2.7%, 5/185, Table 6) had voice change, which gradually and spontaneously recovered within 3 months (minor complications, SIR classification A). No delayed or life-threatening complications occurred during the follow-up.

**Table 6:** Complications of RFA during the follow-up.

Complications	Values, *n* (%)
Major complications	0 (0)
Permanent voice change (>3 months)	0 (0)
Airway obstruction	0 (0)
Infection	0 (0)
Permanent hypoparathyroidism (>3 months)	0 (0)
Rupture of the ablated nodules	0 (0)
Minor complications	5 (2.7)
Transient voice change (≤3 months)	5 (2.7)
Hematoma	0 (0)
Transient hypoparathyroidism (≤3 months)	0 (0)

As for the thyroid function, the thyroid-stimulating hormone (TSH) level changed from 1.12 μIU/mld (range: 0.01–6.39 μIU/mld) at baseline to 1.39 μIU/mld (range: 0.01–8.87 μIU/mld) at the endpoint, and the number of TSH abnormalities changed from 12 patients (6.5%) to 5 patients (2.7%).

## Discussion

Our results demonstrated that MNG is positively affected by RFA at a mean follow-up time of 22 months with an obvious and long-lasting shrinkage of dominant nodules, significant improvements in cosmetic and symptom scores, a low incidence of complications, and a 2.2% of retreatment rate due to the progression of all nodule loads after RFA. The major aim of RFA is to eliminate, or at least reduce, clinical discomfort and avoid further invasive treatment. For a disease such as MNG, in which a pathological process involves the whole thyroid gland (24), this discomfort is not only associated with the presence of dominant nodules but also associated with whole thyroid swelling or all nodule burdens. Therefore, a comprehensive assessment of the efficacy of the procedure for the treatment of the entire thyroid should be important and necessary for MNG patients. In recent years, following previous studies of RFA for MNG or retrosternal goiter, although RFA is recognized as an effective procedure for shrinking ablated nodules, with VRRs of approximately 70% to 80% at 6 to 12 months (12, 13, 14, 15), data on the improvement of cosmetic outcomes and the relief of symptoms related to whole thyroid swelling or the presence of all nodule loads in a large sample are still rare. Based on this, we selected 185 MNG patients who underwent a single session of RFA and were followed up for 22.38 ± 13.75 months (range, 12–60 months), and the final results confirmed the persistent elimination of aesthetic and symptomatic concerns, which became significant starting from one month and progressed until reaching the endpoint. Additionally, the size of the ablated dominant nodules was significantly shrunken, with a VRR of 78.47% (range, 17.97%–99.42%) and 89.20% (range, 25.29%–100%) at 6 and 12 months after the procedure, respectively, which was also consistent with the aforementioned studies.

Although the treatment response of the ablated target nodules is a key indicator of treatment effectiveness, the ‘unequivocal progression or appearance’ of non-target or new lesions also denotes a possible trend of the overall level of substantial worsening, which is closely related to continued therapy (25). Early attempts to evaluate the recurrence and retreatment of MNG patients in some previous surgical studies have confirmed that these problems are of great concern, and recurrence was defined as the presence of a nodule larger than or equal to 3 mm or 5 mm on US (3, 23, 26, 27, 28, 29, 30), with studies finding recurrence rates ranging from 2.0% to 60.6% and reoperation rates from 1.6% to 8.0% (25, 26, 27, 28, 29). However, these definitions were not fully applicable to RFA for the following reasons: (i) discrepancies in the definitions of recurrence made it difficult to compare the results among research groups, (ii) recurrence in surgery studies only focused on new nodules, but for RFA, non-target nodules also had to be evaluated; (iii) some 5 mm or 3 mm nodules that were recognized as indicating recurrence were actually ‘non-measurable,’ because the accuracy and reproducibility of the US measurements and the clinical significance could not be guaranteed (25); and (iv) it is debatable whether nodules <1 cm can be defined as a recurrence. To address inconsistencies in the evaluation criteria in solid tumors (25), based on the risk of retreatment, our study first self-defined CR, SD, and PD to assess the development of all nodule burdens, especially non-target and new nodules, after complete ablation of target nodules. CR is defined as the disappearance of all active nodules during the follow-up period. SD suggests the persistent complete necrosis of target nodules without the tendency of worsening of non-target or new lesions. PD is defined as non-target and new nodules showing substantial worsening that requires close surveillance evaluation or possible continued therapy, even for target lesions showing the best treatment response. According to our criteria, the vast majority of patients were in a progression-free state after complete ablation of all dominant nodules by a single session of RFA, and more importantly, the overall progression rate was 9.7% and the rate of need for retreatment due to PD was much lower (2.2%, 4/185), which were not higher than previous surgical studies. This can mainly be attributed to the different minimum nodule size criteria of clinically relevant negative development: contrary to most surgical studies in which a nodule diameter of 5 mm or greater was the criterion for recurrence, our study used a larger nodule diameter as a criterion for PD (1 cm or greater). The main basis for our revision is that only nodules ≥1 cm might have the greater potential to cause clinical discomfort or be clinically significant cancers (4), and it is also supported by many guidelines that recommend against the biopsy of nodules smaller than 1 cm (4, 6, 31). Therefore, we believe that this criterion and the rate of PD may be more consistent with the clinical course and significance of MNG. Meanwhile, to endocrinologists, surgeons, and MNG patients, this also suggests that a single-session RFA may not expose MNG patients to a high retreatment rate after complete ablation of dominant nodules, so that the originally expected need for multiple treatments may not be a reason to limit the clinical application of RFA for MNG.

We should also consider the limitations of this study. First, although all patients had a follow-up of over 12 months, the number of patients showed a progressive reduction over time, particularly after 36 months. The reasons may include the patients skipping the periodic visits due to the complete remission of MNG or other nonclinical causes, such as geographical distance. Further prospective studies are still needed to validate our results. In addition, supporting evidence on whether the number of dominant nodules ablated by a single RFA session affects outcomes remains scarce, but this issue was not explored in our study. The core factors of nodule regrowth cannot be distinguished by routine US, including the difference between ablation zones and recurrent or active nodules. Therefore, a discriminable parameter of regrowth, such as an ablated nodule volume increase of ≥50%, was used in this study. Finally, different ablation volumes of target nodules may have a major impact on the outcome of RFA for MNG, but only patients with completely ablated dominant nodules were included in this study. Therefore, our results have a limited scope of application and may not be generalized to all institutions.

In summary, for selected MNG patients with 2–5 benign nodules in the thyroid, more than 50% normal thyroid tissue and well-defined target dominant nodules that were completely ablated in a single session of RFA, the current study showed that RFA has a reasonable effect in that it significantly improves the cosmetic outcomes and symptoms, effectively shrinks dominant nodules, and has low rates of PD and retreatment based on all nodule burdens.

## Declaration of interest

The authors declare that there is no conflict of interest that could be perceived as prejudicing the impartiality of the study reported.

## Funding

This work was supported by the fifth Five-Year Project of the Third Affiliated Hospital of Sun Yat-Sen University (grant 2023WW102), the 5010 Clinical Research Project of Sun Yat-sen Universityhttp://dx.doi.org/10.13039/501100002402 (grant 2022010), the Key Scientific and Technological Program of Guangzhou City (grant 201802020023), the Natural Science Foundation of Guangdong Provincehttp://dx.doi.org/10.13039/501100003453 (grant 2020A1515010425), the National Natural Science Foundation of Chinahttp://dx.doi.org/10.13039/501100001809 (grant 81971632), the Key Scientific and Technological Projects of Guangdong Province (grant 2019B020235002), and the Key Scientific and Technological Projects of Guangdong Province (grant 2018B030332001-1).

## Author contribution statement

RG contributed to literature research, data collection, formal analysis, and manuscript drafting. BZ contributed to clinical studies, formal analysis, and manuscript drafting. TW contributed to clinical studies and statistical analysis. YL contributed to clinical studies. TY contributed to statistical analysis. YH contributed to data collection. JQ contributed to data collection. ZY, WX, and JR contributed to study design and manuscript editing, and JR is the guarantor of integrity of the entire study. All authors provided clinical feedback in interpreting the results, contributed critically to subsequent revisions, and approved the final version of the manuscript.
